# Finite element analysis of coronoid prostheses with different fixation methods in the treatment of comminuted coronoid process fracture

**DOI:** 10.1186/s10195-022-00675-2

**Published:** 2022-12-05

**Authors:** Hailong Zhang, Kun-Jhih Lin, Po-Yi Liu, Yi Lu

**Affiliations:** 1 Department of Sports Medicine, Beijing Jishuitan Hospital, No. 31 Xinjiekou East Street, Xicheng, Beijing, 100035 China; 2 Department of Electrical Engineering & Translation Technology Center for Medical Device, Chung Yuan Christian University, Taoyuan, Taiwan China; 3 Department of Sports Medicine, Kaohsiung Medical University, Kaohsiung, Taiwan China

**Keywords:** Coronoid, Prosthesis, Fixation, Finite element analysis

## Abstract

**Background:**

Comminuted fractures of the coronoid process significantly compromise the stability and function of the elbow joint. Reconstruction of the coronoid process with a prosthesis has been suggested as an alternative to restore the architecture. The purpose of this study was to investigate the strength and stability of various methods for the fixation of a coronoid prosthesis by finite element analysis.

**Materials and methods:**

A coronoid prosthesis was designed based on the morphological information from computed tomography images acquired from 64 subjects in whom the top 40% of the coronoid process height was replaced. Four methods for the fixation of the prosthesis were suggested: (1) a double 2.0-mm fixation bolt, anterior to posterior; (2) a double 2.5-mm fixation bolt, anterior to posterior; (3) a single 4.0-mm fixation bolt, posterior to anterior; (4) a single 4.5-mm fixation bolt, posterior to anterior. The integrated prosthesis-bone constructs were analyzed via the finite element analysis of 10 simulated proximal ulna models with loading applied along the axis of the humerus and with three different elbow flexion angles (30°, 90°, and 130°). The maximum principal stress and the total deformation were quantified and compared.

**Results:**

A coronoid prosthesis was developed. The maximum principal stress of the fixation bolts occurred around the neck of the fixation bolt. For a comparison of the strengths of the four fixation methods, the maximum principal stress was the lowest for fixation using a single 4.5-mm fixation bolt. The value of the maximum principal stress significantly decreased with increased elbow flexion angle for all fixation methods. The maximum deformation of the fixation bolts occurred at the head of the fixation bolt. For a comparison of the maximum deformations in the four fixation methods, the maximum deformation was the lowest for fixation using a single 4.5-mm fixation bolt. The value of the maximum deformation significantly decreased with increased elbow flexion angle for all fixation methods.

**Conclusions:**

The present study suggested that fixation of a coronoid prosthesis with a single 4.5-mm fixation bolt from posterior to anterior is an excellent option in terms of the strength and stability.

*Level of Evidence* Experimental study.

## Introduction

The role of the coronoid process in the stability of the elbow joint has been well established. Clinical and biomechanical evidence has suggested that it functions as the paramount stabilizer, preventing posterior dislocation and resisting rotational instability [1–3]. Fractures of the coronoid process, especially the comminuted pattern commonly seen in terrible triad and trans-olecranon fractures or other injuries, result in persistent instability of the elbow and significantly jeopardize activities of daily life [4]. Numerous methods have been developed for reconstructing the coronoid process using auto- or allograft. However, the resulting outcomes are unpredictable, with donor site comorbidities reported [5–8].

The application of a prosthesis in treating comminuted coronoid fracture was first described in 2017, when O’Driscoll developed the concept for the design of the coronoid prosthesis and demonstrated promising results in three cases [9]. After that, the coronoid prosthesis received attention, as it allowed better restoration of the elbow’s integrity while avoiding the pitfall of grafting [9]. Just as it is for the prostheses of other joints, the longevity of the prosthesis is a concern, as it is closely associated with the long-term clinical outcome, while the most important factor in the longevity of the prosthesis is the fixation method used. It is believed that a proper fixation method is of great significance to the promotion of osseous integration, which, in turn, reduces the risk of breakage and aseptic loosening of the prosthesis [10, 11].

Studies on the effectiveness and biomechanical behavior of the screw fixation of other joints have been extensively carried out [12–14]. In terms of a coronoid prosthesis, only a few investigations have examined this specific issue [9, 15, 16]. To date, only one study has discussed this question by comparing the effects of three fixation methods in minimizing the micro-motion of the prosthesis. These methods included press-fit cementless fixation, screw fixation, and cement fixation, and the results suggested that screw fixation may be sufficient to allow osseous integration to be achieved for long-term fixation [15]. However, the biomechanical behavior of screw fixation in the study was only investigated in the setting of two 2.4-mm cortical screws. Comparison of the biomechanical behavior of coronoid prostheses with different fixation methods has rarely been investigated, and it is still unknown whether other fixation methods would result in better stability.

The purpose of this finite element analysis (FEA) study was to investigate the strength of the fixation bolts and the stability of fixation achieved with various fixation methods at different elbow flexion angles. The hypothesis was that prosthesis fixation with a single fixation bolt 4.5 mm in diameter, which was fixed from posterior to anterior, shows better strength and fixation stability compared with other methods.

## Materials and methods

### 3D model of the proximal ulna

**Fig. 1:**
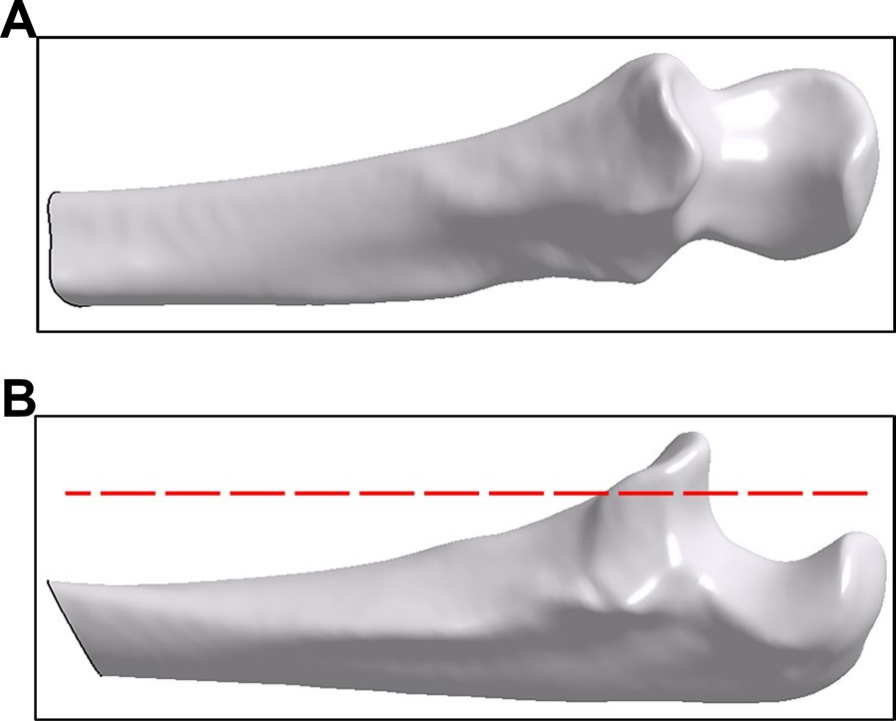
3D ulna model reconstruction and simulation of coronoid process fracture. **A** A 3D model of the ulna was reconstructed based on CT images. **B** A cutting plane (*dashed red line*) trimmed the upper 40% of the coronoid process for the simulation of coronoid process fracture, and the upper segment was used as the model when designing the coronoid prosthesis

The study was ethically approved by the institutional review board, and informed consent was obtained from the patients. Computed tomography (CT) scans (Aquillion 64, Toshiba America Medical Systems, Tustin, CA, USA) of right elbows without fracture, deformity, and osteoarthritis from 10 normal subjects (5 females and 5 males; 38 ± 6.6 y) were acquired. The CT scans were performed with a resolution of 512 × 512 pixels and a slice thickness of 0.8 mm using the standard 120-kVp and 200-mA bone reconstruction sequence. Three-dimensional (3D) proximal ulna reconstruction was achieved from the CT slice images using PTC Creo 2.0 software after the cortical and cancellous contours were outlined based on the grayscale differences in the CT images (Fig. 1A). A cutting plane trimmed the upper 40% of the coronoid process to generate 10 models to simulate coronoid process fracture (Fig. 1B) [7, 17].

### Design of the model of the coronoid prosthesis

**Fig. 2:**
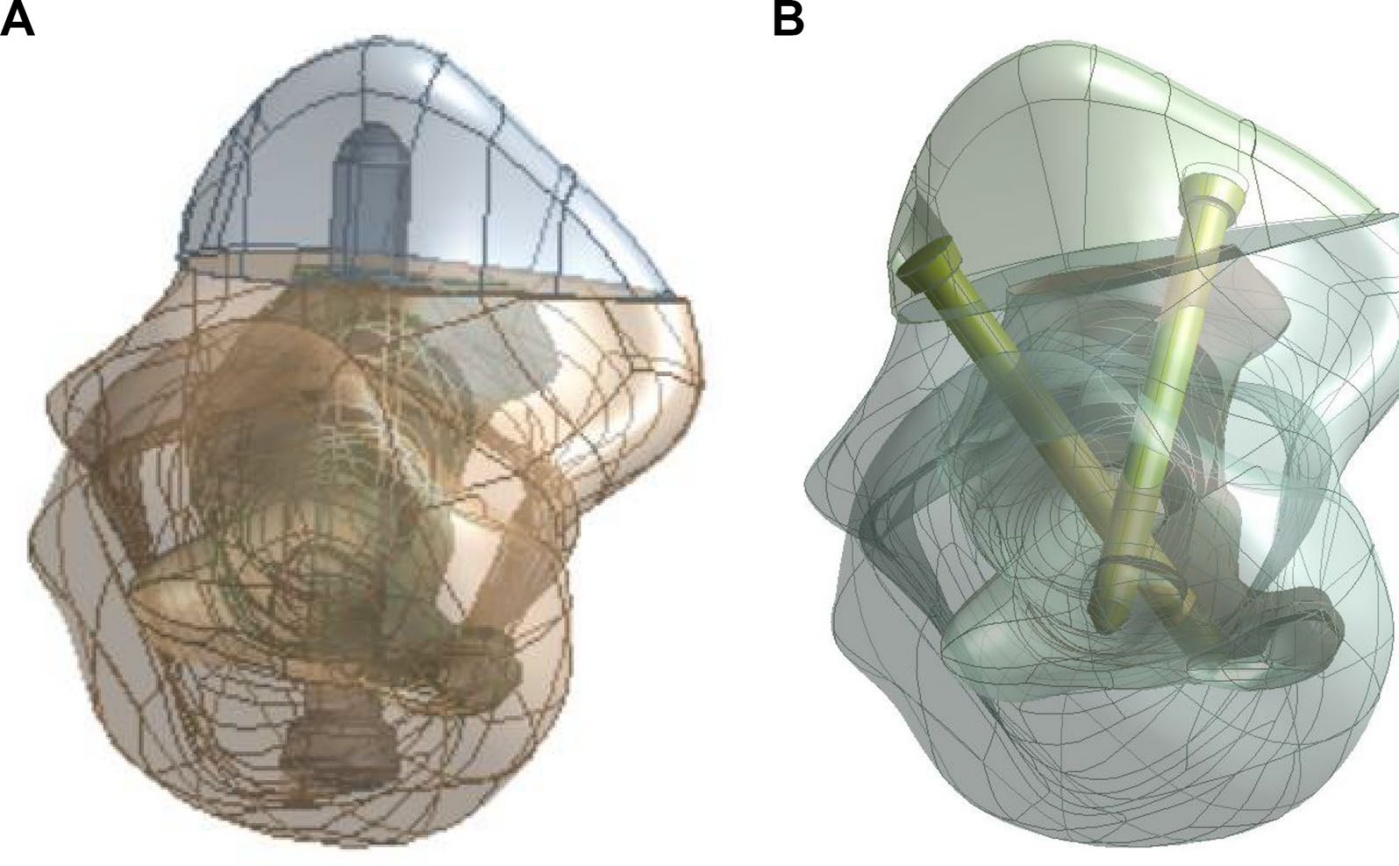
Fixation of the coronoid prosthesis with a single or double bolt. **A** Fixation of the coronoid prosthesis with a single bolt; the direction of the bolt is perpendicular to the bone–prosthesis interface from posterior to anterior. **B** Fixation of the coronoid prosthesis with a double bolt; the direction of the screw is 60° to the bone–prosthesis interface in the coronal plane from anterior to posterior

To generate a coronoid prosthesis, CT scans of right elbows without fracture, deformity, and osteoarthritis from 64 normal subjects (23 females and 41 males; 41.4 ± 9.7 y) were acquired using the sequence mentioned above. An anatomically shaped coronoid prosthesis was designed to replace 40% of the coronoid process height, which was shown in the literature to be a fracture height commonly encountered clinically [7, 17]. Measurements included the coronoid process height, the width, the radius of the lateral facet of the coronoid process, and the radius of the medial facet of the coronoid process. The material of the prosthesis was designated as Ti6Al4V. Four fixation methods for the prosthesis were analyzed for a comparison of the fixation performance: (1) a double 2.0-mm fixation bolt; (2) a double 2.5-mm fixation bolt; (3) a single 4.0-mm fixation bolt; and (4) a single 4.5-mm fixation bolt. For the fixation methods with a single fixation bolt, the direction of the fixation bolt was perpendicular to the bone–prosthesis interface from posterior to anterior; for the fixation methods with a double fixation bolt, the direction of the fixation bolt was 60° to the bone–prosthesis interface in the coronal plane from anterior medial and anterior lateral to posterior (Fig. 2). The fixation bolt models were generated using CAD software (PTC Creo 2.0, Parametric Technologies Corp., Needham, MA, USA).

### Finite element analysis

For each model, four integrated prosthesis–bone constructs were imported into a commercial FEA solver, ANSYS Workbench 19 (ANSYS Inc., Canonsburg, PA, USA), for computational analysis. Due to the complex geometry of the fixation bolt thread, the bolt shaft was simplified to a cylinder and the mesh consisted of tetrahedral elements arranged in a linear fashion only. To ensure that the numerical values produced by the analysis reached convergence, a convergence test of total strain energy was performed, and mesh convergence was tested by increasing the size of the mesh elements in six steps from size 9 to size 4. For the convergence test, a new FEA model with more elements was calculated and compared to the presented FEA model. The total strain energy of the whole model and characteristic points with the same position in various mesh groups were then selected, and the vertical displacements of the nodes in these groups were retrieved. The results of the convergence tests were compared to the results of the group with a mesh size of 3 mm. Errors for all groups were within an acceptable range (< 0.3%), which suggested that all mesh groups were convergent. The current study used size 6 as the mesh planning element size, and the mesh generated for each design contained 57321 elements on average.

Homogeneity and isotropic linearity was assumed for all components in the current study. To assign the mechanical properties of the cancellous bone, the bone density and the elastic modulus were calculated based on the CT Hounsfield unit (HU) and the following equations:

$$ \rho = 131000\; + \;1067HU $$

$$ E = \;6850e^{1.49}, $$

where* ρ* is the apparent density (g/cm^3^) and* E* is the elastic modulus (MPa). The elastic modulus values of the cortical bone and prosthesis were assigned to be 12 GPa and 110 GPa, respectively, while the Poisson ratios of the cortical bone and the prosthesis were both assigned to be 0.3 [6, 18, 19]. The base of the ulna was fixed in all degrees of freedom. Full constraints were applied between the fixation bolts and surrounding bone and between the implant and the bolt head to simulate the tightened locking [20].

**Fig. 3:**
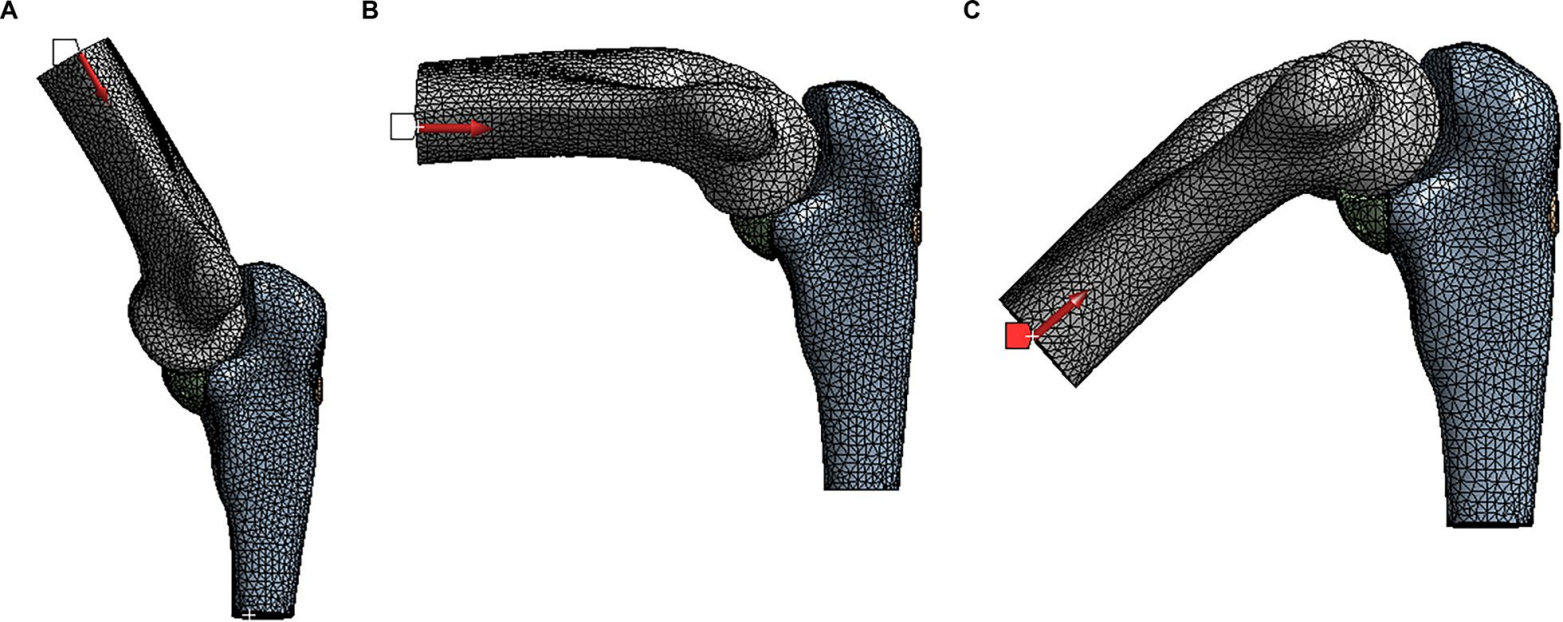
Loading at various elbow flexion angles. **A** Loading at 30° elbow flexion. **B** Loading at 90° elbow flexion. **C** Loading at 130° elbow flexion. The* red arrow* indicates the direction of the loading, which is along the axis of the humerus. The* triangles* represent the fixed end

For the loading conditions on the model in each group, a loading of 490.3 N at three flexion angles (30°, 90°, and 130°) was applied along the axis of the humerus to mimic the injury mechanism where patients fall down with a hand supporting (Fig. 3) [21]. A total of 120 FE models were tested using combinations of four different fixation methods and three different flexion angles. The maximum principal stress and the total deformation around the fixation bolt were investigated.

### Statistics

The values are expressed as the mean and standard deviation (SD). For a comparison of the maximal principal stress on and the maximum deformation of the bolt attained with different fixation methods, one-way analysis of variance (ANOVA) was used, while the post hoc Tukey’s honestly significant difference (HSD) test was used to compare the maximum principal stress and the maximum deformation among groups, if present. For the investigation of the effect of flexion angle on the maximal principal stress on and the maximum deformation of the bolt, linear regression analysis was performed. A* p* value of < 0.05 was considered significantly different, while* α* and* β* were set to be 0.05 and 0.2 in the calculation of sample size. The total of 10 CT scans performed in this study were enough to generate a power of > 80% for the comparison.

## Results

### Measurement of the coronoid process

To design the coronoid process, the height, length, and width as well as the radius of the medial facet and those of the lateral facet of the upper 40% of the coronoid process were measured. The height of the target fragment was 12.94 ± 2.14 mm. The length of the target fragment was 24.11 ± 2.23 mm. The width of the target fragment was 22.98 ± 2.14 mm. The radii of the medial and lateral facets were 6.11 ± 1.34 mm and 11.97 ± 3.82 mm, respectively.

### Distribution of stress on the fixation bolt

**Fig. 4:**
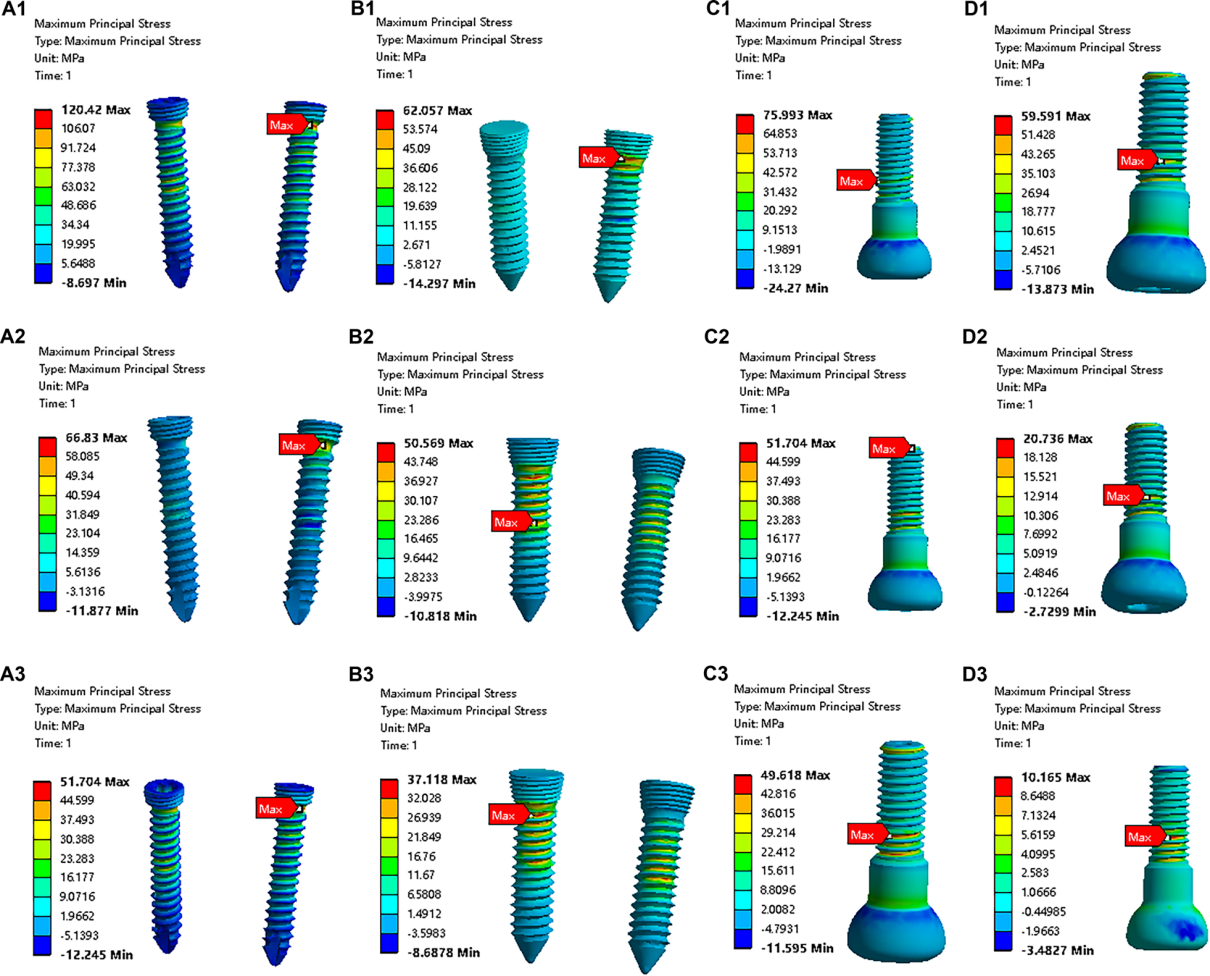
The stress distribution on the fixation bolt. **A1** The stress distribution on the fixation bolts when using a double 2.0-mm bolt at 30° elbow flexion. **A2**. The stress distribution on the fixation bolts when using a double 2.0-mm bolt at 90° elbow flexion. **A3** The stress distribution on the fixation bolts when using a double 2.0-mm bolt at 130° elbow flexion. **B1** The stress distribution on the fixation bolts when using a double 2.5-mm bolt at 30° elbow flexion. **B2** The stress distribution on the fixation bolts when using a double 2.5-mm bolt at 90° elbow flexion. **B3**. The stress distribution on the fixation bolts when using a double 2.5-mm bolt at 130° elbow flexion. **C1** The stress distribution on the fixation bolt when using a single 4.0-mm bolt at 30° elbow flexion. **C2** The stress distribution on the fixation bolt when using a single 4.0-mm bolt at 90° elbow flexion. **C3** The stress distribution on the fixation bolt when using a single 4.0-mm bolt at 130° elbow flexion. **D1** The stress distribution on the fixation bolt when using a single 4.5-mm bolt at 30° elbow flexion. **D2** The stress distribution on the fixation bolt when using a single 4.5-mm bolt at 90° elbow flexion. **D3** The stress distribution on the fixation bolt when using a single 4.5-mm bolt at 130° elbow flexion

The maximum principal stress on the fixation bolts was calculated and was found to occur around the neck of the fixation bolt (Table 1) (Fig. 4). For a comparison of the strengths of the four fixation methods, the maximum principal stress differed significantly among groups (at 30° flexion, *F*(3, 35) = 190.5, *P* < 0.0001; for a 90° flexion angle, *F*(3, 35) = 185.1, *P* < 0.0001; for a 130° flexion angle, *F*(3, 35) = 164.2, *P* < 0.0001). The post hoc Tukey’s HSD test showed that the maximum principal stress that occurred when using a single 4.5-mm fixation bolt was significantly smaller than the maximum principal stress that occurred in the other groups (Fig. 6A–C) (at 30° flexion, for a comparison between the double 2.0-mm fixation bolt and the single 4.5-mm fixation bolt, *P* < 0.0001; for a comparison between the double 2.5-mm fixation bolt and the single 4.5-mm fixation bolt, *P* = 0.7761; for a comparison between the single 4.0-mm fixation bolt and the single 4.5-mm fixation bolt, *P* < 0.0001; at 90° flexion, for a comparison between the double 2.0-mm fixation bolt and the single 4.5-mm fixation bolt, *P* < 0.0001; for a comparison between the double 2.5-mm fixation bolt and the single 4.5-mm fixation bolt, *P* < 0.0001; for a comparison between the single 4.0-mm fixation bolt and the single 4.5-mm fixation bolt, *P* < 0.0001; at 130° flexion, for a comparison between the double 2.0-mm fixation bolt and the single 4.5-mm fixation bolt, *P* < 0.0001; for a comparison between the double 2.5-mm fixation bolt and the single 4.5-mm fixation bolt, *P* < 0.0001; for a comparison between the single 4.0-mm fixation bolt and the single 4.5-mm fixation bolt, *P* < 0.0001). In terms of the strength with various flexion angles, the decrease in the maximum principal stress was significantly correlated with the increase in the elbow flexion angle for all fixation methods (Fig. 7A–D) (for fixation using the double 2.0-mm fixation bolt, *P* < 0.0001; for fixation using the double 2.5-mm fixation bolt, *P* < 0.0001; for fixation using the single 4.0-mm fixation bolt, *P* < 0.0001; for fixation using the single 4.5-mm fixation bolt, *P* < 0.0001).

**Table 1:** Comparing the distribution of maximal principal stress on the fixation bolt between groups

Group	Flexion at 30° (MPa)	Flexion at 90° (MPa)	Flexion at 130° (MPa)
Double 2.0-mm bolt	124.03 ± 10.11	66.83 ± 4.72	51.86 ± 4.36
Double 2.5-mm bolt	62.24 ± 5.29	50.80 ± 4.93	37.15 ± 4.99
Single 4.0-mm bolt	75.91 ± 5.83	54.18 ± 4.95	43.90 ± 5.10
Single 4.5-mm bolt	59.25 ± 4.51	20.08 ± 3.77	10.35 ± 3.10

### Distribution of the total deformation of the fixation bolt

**Fig. 5:**
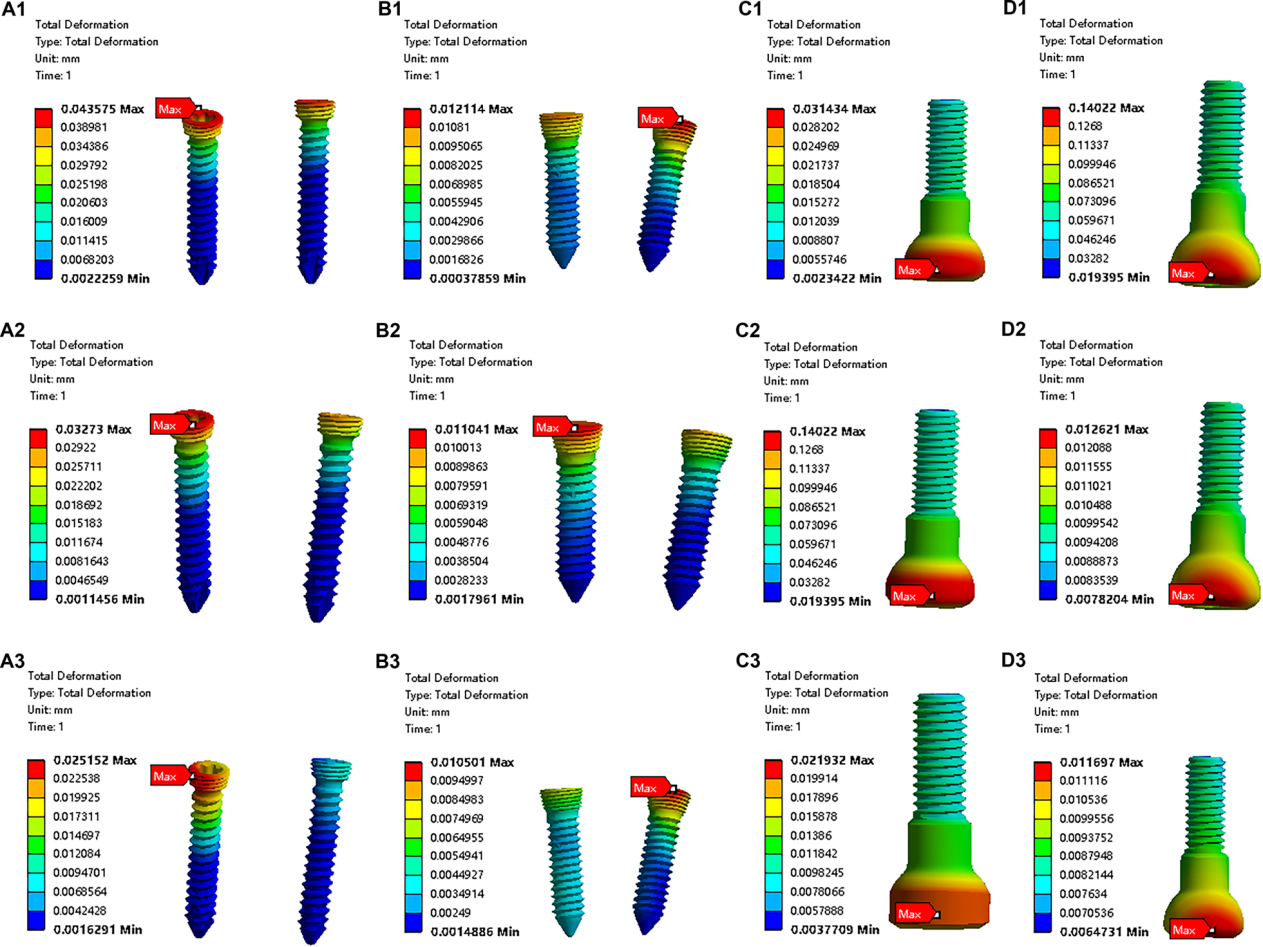
The total deformation of the fixation bolt. **A1** The total deformation of the fixation bolts when using a double 2.0-mm bolt at 30° elbow flexion. **A2** The total deformation of the fixation bolts when using a double 2.0-mm bolt at 90° elbow flexion. **A3** The total deformation of the fixation bolts when using a double 2.0-mm bolt at 130° elbow flexion. **B1** The total deformation of the fixation bolts when using a double 2.5-mm bolt at 30° elbow flexion. **B2** The total deformation of the fixation bolts when using a double 2.5-mm bolt at 90° elbow flexion. **B3** The total deformation of the fixation bolts when using a double 2.5-mm bolt at 130° elbow flexion. **C1** The total deformation of the fixation bolt when using a single 4.0-mm bolt at 30° elbow flexion. **C2** The total deformation of the fixation bolt when using a single 4.0-mm bolt at 90° elbow flexion. **C3** The total deformation of the fixation bolt when using a single 4.0-mm bolt at 130° elbow flexion. **D1** The total deformation of the fixation bolt when using a single 4.5-mm bolt at 30° elbow flexion. **D2** The total deformation of the fixation bolt when using a single 4.5-mm bolt at 90° elbow flexion.** D3** The total deformation of the fixation bolt when using a single 4.5-mm bolt at 130° elbow flexion

**Fig. 6:**
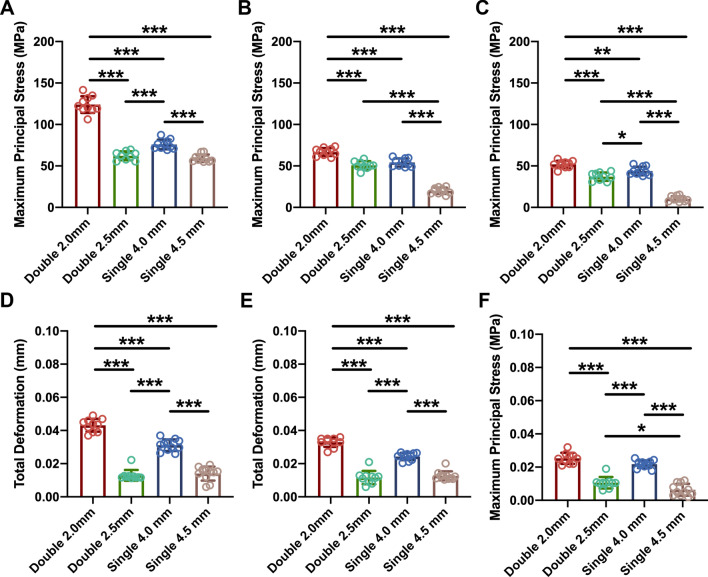
Comparison of the maximum principal stress and the maximum deformation obtained with different fixation patterns. **A**, **D** The maximum principal stress and the maximum deformation were both smallest when using a single 4.5-mm fixation bolt at 30° flexion. **B**, **E** The maximum principal stress and the maximum deformation were both smallest when using a single 4.5-mm fixation bolt at 90° flexion. **C**, **F** The maximum principal stress and the maximum deformation were both smallest when using a single 4.5-mm fixation bolt at 130° flexion. Data shown are the mean and SD. *n * = 10; *p < 0.05, **p < 0.01, ***p < 0.001

**Fig. 7:**
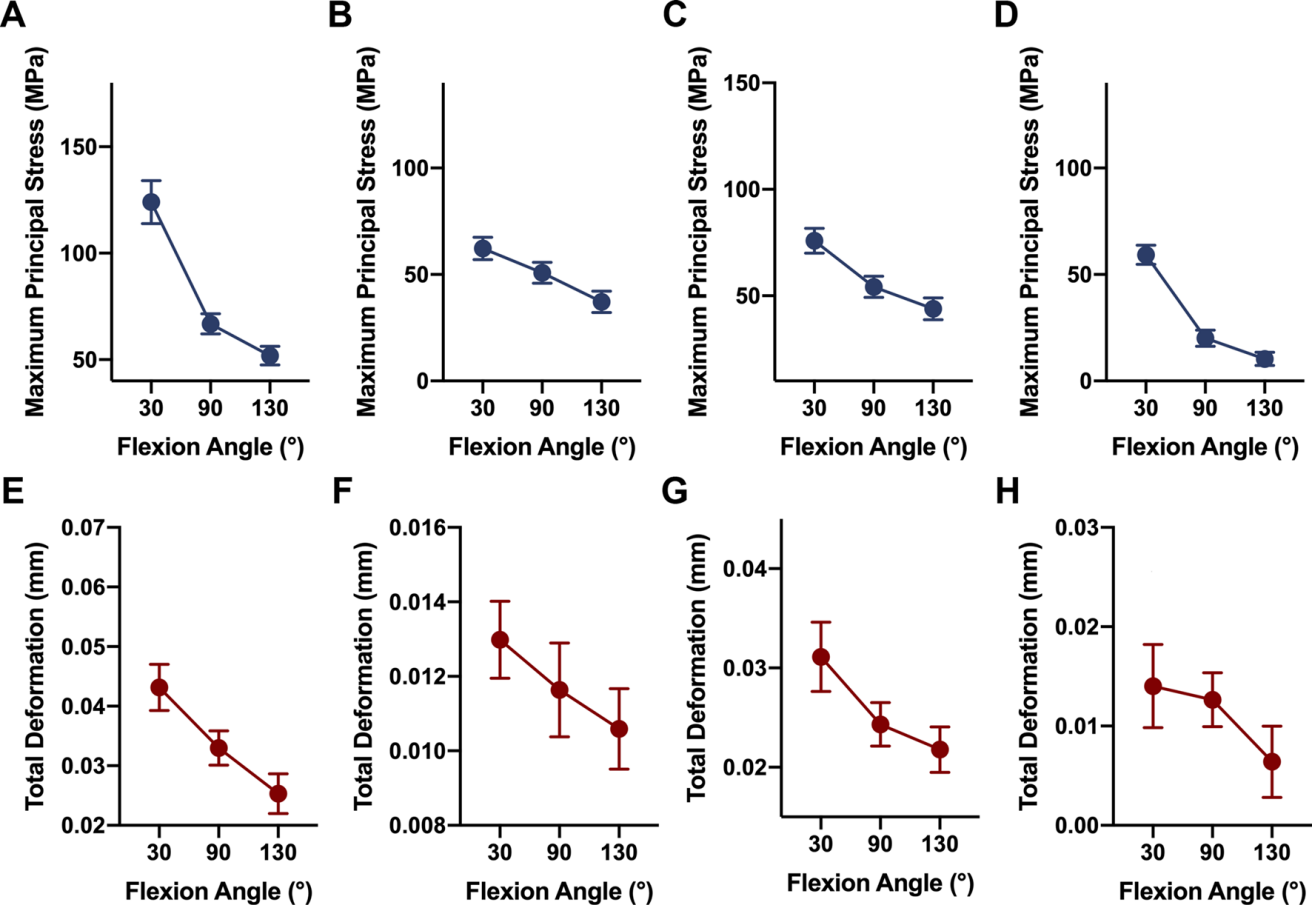
The effect of flexion angle on the maximum principal stress and the maximum deformation. **A**, **E** The maximum principal stress and the maximum deformation both significantly decrease as the elbow flexion angle increases when using a double 2.0-mm fixation bolt. **B**, **F** The maximum principal stress and the maximum deformation both significantly decrease as the elbow flexion angle increases when using a double 2.5-mm fixation bolt. **C**, **G** The maximum principal stress and the maximum deformation both significantly decrease as the elbow flexion angle increases when using a single 4.0-mm fixation bolt. **D**, **H** The maximum principal stress and the maximum deformation both significantly decrease as the elbow flexion angle increases when using a single 4.5-mm fixation bolt. Data shown are the mean and SD. *n* = 10

The maximum deformation of the fixation bolt was calculated and was found to occur at the head of the fixation bolt (Table 2) (Fig. 5). For a comparison of the total deformation in the four fixation methods, the maximum total deformation differed significantly among groups (at 30° flexion, *F*(3, 35) = 155.4, *P* < 0.0001; for a 90° flexion angle, *F*(3, 35) = 118.9, *P* < 0.0001; for a 130° flexion angle, *F*(3, 35) = 83.34, *P* < 0.0001). The post hoc Tukey’s HSD test showed that the maximum total deformation that occurred when using a single 4.5-mm fixation bolt was significantly smaller than the maximum total deformation that occurred in the other groups (Fig. 6D–F) (at 30° flexion, for a comparison between a double 2.0-mm fixation bolt and a single 4.5-mm fixation bolt, *P* < 0.0001; for a comparison between a double 2.5-mm fixation bolt and a single 4.5-mm fixation bolt, *P* = 0.9191; for a comparison between a single 4.0-mm fixation bolt and a single 4.5-mm fixation bolt, *P* < 0.0001; at 90° flexion, for a comparison between a double 2.0-mm fixation bolt and a single 4.5-mm fixation bolt, *P* < 0.0001; for a comparison between a double 2.5-mm fixation bolt and a single 4.5-mm fixation bolt, *P* = 0.8645; for a comparison between a single 4.0-mm fixation bolt and a single 4.5-mm fixation bolt, *P* < 0.0001; at 130° flexion, for a comparison between a double 2.0-mm fixation bolt and a single 4.5-mm fixation bolt, *P* < 0.0001; for a comparison between a double 2.5-mm fixation bolt and a single 4.5-mm fixation bolt, *P* = 0.0233; for a comparison between a single 4.0-mm fixation bolt and a single 4.5-mm fixation bolt, *P* < 0.0001). In terms of the total deformation with various flexion angles, the decrease in the maximum total deformation was significantly correlated with the increase in the elbow flexion angle for all fixation methods (Fig. 7E–H) (for fixation using a double 2.0-mm fixation bolt, *P* < 0.0001; for fixation using a double 2.5-mm fixation bolt, *P* < 0.0001; for fixation using a single 4.0-mm fixation bolt, *P* < 0.0001; for fixation using a single 4.5-mm fixation bolt, *P* < 0.0001).

**Table 2:** Comparing the distribution of total deformation of the fixation bolt between groups

Group	Flexion 30° (mm)	Flexion 90° (mm)	Flexion 130° (mm)
Double 2.0-mm bolt	0.043 ± 0.0039	0.033 ± 0.0029	0.025 ± 0.0033
Double 2.5-mm bolt	0.013 ± 0.0033	0.012 ± 0.0040	0.011 ± 0.0034
Single 4.0-mm bolt	0.031 ± 0.0035	0.024 ± 0.0022	0.022 ± 0.0023
Single 4.5-mm bolt	0.014 ± 0.0042	0.013 ± 0.0027	0.0064 ± 0.0036

## Discussion

In this study, a coronoid prosthesis was developed based on the morphology of the upper 40% of the coronoid process, and a simulated model with a coronoid process defect that was reconstructed by the coronoid prosthesis was analyzed using a finite element analysis to investigate the fixation strength of the prosthesis when applying different fixation methods at various elbow flexion angles. As hypothesized, prosthesis fixation with a single bolt 4.5 mm in diameter fixed from posterior to anterior showed better strength and fixation stability.

The coronoid process of the ulna is the key element in the stability of the elbow joint [1]. In the case of a comminuted coronoid process fracture, when the reduction and fixation of the fracture fragment could not be achieved, reconstruction using autograft or allograft was regarded as a choice [5, 6, 8]. However, these methods were associated with either donor site comorbidity or limited availability [5, 6, 8]. Recently, the coronoid prosthesis design was developed to better restore the elbow stability. Alia pointed out that a coronoid prosthesis may be a feasible treatment option to restore elbow stability, based on a biomechanical study [15]. O’Driscoll and colleagues reported the follow-up of three cases of comminuted coronoid process fracture treated with a coronoid prosthesis and suggested that this treatment provided an excellent outcome. It was believed that a coronoid prosthesis may offer a promising solution for a comminuted coronoid process fracture. But, to date, no further studies that follow on from those results have been conducted. One reason for this may be the poor understanding of how to maintain the long-term stability of the coronoid prosthesis [9].

The most common reason for revision surgery for a prosthesis other than a coronoid prosthesis in clinical practice is aseptic loosening, which is associated with an unexpected cost and a poor clinical outcome. The most important measure that can diminish this complication is to optimize the design of the fixation method. A previous study examined three fixation methods in terms of minimizing the micro-motion of the prosthesis, including press-fit cementless fixation, screw fixation, and cement fixation, and the results suggested that screw fixation may benefit osseous integration [15]. However, the biomechanical behavior in the condition with screw fixation was only investigated using two 2.4-mm cortical screws. So far, no results for other fixation patterns have been reported in the literature.

Methods for investigating the biomechanical behavior of the prosthesis can be categorized into biomechanical experimentation and computational simulation. Biomechanical experimentation can offer parameters from human material with an actual load, but it is limited by the availability of human tissue specimens and patient variability, which results in a small sample size and underpowered statistical significance [22]. Computational simulation—particularly FEA of a 3D model reconstructed from CT images, which allows the simulation of multiple loading parameters and the modification of boundary conditions—has been accepted as a powerful tool for designing a prosthesis and evaluating the effects of various prosthesis fixation methods [22].

Based on our previous study, we studied four fixation methods and examined their strength and stability by finite element analysis. The results indicated that the maximal principal stress and the maximum deformation are smallest when using a single 4.5-mm bolt for fixation at all three flexion angles, whereas it was largest with double 2.0-mm bolt fixation. These results suggest that the diameter of the fixation bolt plays an important role in resisting micro-motion of the interface between the prosthesis and the bone. The larger diameter demonstrated better biomechanical characteristics, but using a larger diameter leads to more volume being occupied by the bolts in the host bone, which compromises bone integrity and increases the risk of fracture. For a comparison of the maximum principal stress on the fixation bolt at different flexion angles, the largest value occurred at 30° flexion, while the smallest value occurred at 130° flexion. It was speculated that the loading generated the greatest shear force on the fixation bolt at 30° flexion, while the loading was directed away from the fixation bolts at 90° and 130° flexion; thus, the maximum principal stress was significantly reduced at these two flexion angles.

Fatigue of the fixation bolt is another reason for prosthesis revision surgery, as repetitive cycle loading on the prosthesis or the fixation device would eventually reach the failure limit. Zand’s biomechanical study suggested that the most common location of bolt fatigue was the root of the thread at the interface between the plate and the bone [23]. Li’s investigation using a femoral neck fracture model indicated that the peak von Mises stress on the screws was concentrated in the middle surface of the screw near the fracture line [24]. In this study, the maximum principal stress was located around the screw neck, which is consistent with a previous study, and it suggested that the maximum principal stress on the fixation bolt occurs at the interface between the bone and the prosthesis. As for the maximum deformity, it was located at the head of the bolt, which meant that the maximum deformity occurred at the far end of the fixation.

In terms of the direction of the bolt for fixation, few reports investigating the role of the direction have been published. In this study, fixation with both single and double bolts was adopted. From a practical perspective, usually medial and lateral approaches to the elbow have been used during the operation to evaluate the coronoid process defect and prosthesis fixation. If double-bolt fixation is employed, the two bolts can be inserted through medial and lateral approaches from anterior to posterior in a divergent manner. When a single bolt is used for fixation, the direction of the bolt should be perpendicular to the bone–prosthesis interface to maximize the stability. If insertion is implemented from anterior to posterior, an extra anterior approach is required to achieve this goal, but this may compromise the neurovascular structure in clinical practice and lead to severe complications. Thus, for single-bolt fixation, a small incision was made at the dorsal part of the ulna, as there is no important neurovascular structure within this region, so the bolt could be inserted from posterior to anterior safely.

There are some limitations of this study. First, the complicated physiologic force loaded on the proximal ulna during movement of the elbow was simplified, but in this FEA model, only axial loading along the humerus was simulated to mimic the force between the trochlea and proximal ulna. The reason for this is that the most common scenario for coronoid process fracture is when patients fall down with the hand supporting and the force is mainly transmitted along the axis of the humerus. The second limitation is that the FEA model was only performed on 10 specimens. A larger sample size is needed to investigate the association between the greater stress in the fixation bolts at low-flexion-angle loading and the corresponding increase in implant displacement. The third limitation of this study is that the structures of the bone and prosthesis were considered isotropic, homogeneous, and linearly elastic, which is not consistent with anatomical and biomechanical reality. The fourth limitation is that, in some of the cases, the fixation bolts would fail in the host bone, which would require further analysis to examine the maximal principal stress in the host bone. The fifth limitation is that the fixation bolt thread was simplified as the thread may introduce stress raisers, and further study is needed to look into this effect.

## Conclusions

The present study suggested that a single 4.5-mm bolt from posterior to anterior is an excellent option for fixation of a coronoid prosthesis in terms of the strength and fixation stability.

## Data Availability

The datasets used during the current study are available from the corresponding author on reasonable request.

## Abbreviations

FEA: Finite element analysis
3D: Three-dimensional
CT: Computed tomography
SD: Standard deviation
ANOVA: Analysis of variance
HSD: Honestly significant difference
